# Seasonal Occurrence, Population Diversity, and Mobilome Features of Environmental *Vibrio parahaemolyticus* in Coastal and Estuarine Waters of Haiyan, Zhejiang, China

**DOI:** 10.3390/microorganisms14081846

**Published:** 2026-08-20

**Authors:** Jingyu Xu, Yangang He, Peiyan He

**Affiliations:** 1 Haiyan Center for Disease Control and Prevention, Jiaxing 314300, China; 13857327399@139.com (J.X.); heyangang12@163.com (Y.H.); 2 Jiaxing Key Laboratory of Pathogenic Microbiology, Jiaxing Center for Disease Control and Prevention, Jiaxing 314050, China

**Keywords:** *Vibrio parahaemolyticus*, environmental surveillance, mobilome, VpaIntIA, super-integron, antimicrobial resistance, T3SS1, seasonal dynamics

## Abstract

*Vibrio parahaemolyticus* is a leading cause of seafood-associated gastroenteritis, yet the ecological and genomic significance of environmental populations as potential reservoirs remains incompletely characterized. From May to October 2024, 108 water samples were collected monthly at one coastal and two estuarine sites in Haiyan, Zhejiang. Confirmed isolates (*n* = 39) underwent whole-genome sequencing, MLST/cgMLST, virulence and AMR gene screening, integron characterization with BLASTp (+2.17.0) integrase family typing, and viral-region prediction. Culture-based detection was absent in May and rose to 66.7% in October (Cochran–Armitage trend, *p* = 0.003), with descriptively higher detection at the coastal site. MLST identified 28 STs including four novel types (Simpson’s diversity = 0.953). All isolates lacked *tdh*, *trh*, and T3SS2 but retained T3SS1 and MAM7. One estuarine isolate carried CALIN-associated *dfrA31* and *qnrVC5*. All integrases matched VpaIntIA (95.9–100% identity), not mobile class 1–3 integrases. Viral regions were detected in 38/39 isolates; filamentous phage annotations in 52.6%. Haiyan coastal *V. parahaemolyticus* shows seasonal and spatial patterns, high diversity, and a mobilome dominated by chromosomal super-integrons, underscoring the need for integrase family typing in environmental surveillance.

## 1. Introduction

*Vibrio parahaemolyticus* is a Gram-negative, halophilic bacterium widely distributed in marine and estuarine environments and recognized as a leading cause of seafood-associated acute gastroenteritis, particularly in Asia [1,2]. Clinical disease is primarily associated with strains harboring the thermostable direct hemolysin (*tdh*) and/or *tdh*-related hemolysin (*trh*) genes, and with two type III secretion systems (T3SSs): T3SS1, present in virtually all strains and contributing to cytotoxicity and autophagy induction, and T3SS2, found predominantly in pandemic clinical strains and functionally linked to *tdh* expression and enterotoxicity [3,4]. The multivalent adhesion molecule MAM7, encoded by *VP1611*, facilitates attachment to host cells and abiotic surfaces and contributes to colonization and environmental persistence [5].

In marine and estuarine environments, *V. parahaemolyticus* abundance is strongly governed by water temperature, with seasonal peaks in warmer months [6,7]. Increasing sea-surface temperatures associated with climate change have been correlated with expanded spatial ranges and prolonged seasonal windows of pathogenic *Vibrio* prevalence in coastal systems worldwide [8]. Beyond temperature, precipitation-driven hydrological inputs—including nutrient loading, salinity modification, and terrestrial microbial influx—are increasingly recognized as co-drivers of *Vibrio* abundance and dispersal in coastal and estuarine environments [9]. Given China’s extensive coastline, high seafood consumption, and intensive aquaculture, environmental surveillance of *V. parahaemolyticus* is an important public health priority [2,10].

While the immediate clinical risk from environmental *V. parahaemolyticus* is often low because most environmental strains lack classical pathogenicity markers, the ecological and genomic significance of environmental populations extends beyond classical virulence. Environmental *V. parahaemolyticus* populations harbor diverse mobile genetic elements (MGEs) that contribute to genomic plasticity and the potential mobilization of AMR and virulence determinants [11,12]. Among these, integrons are particularly relevant. *Vibrio* species possess large chromosomal super-integrons—ancient gene-capture systems characterized by IntIA-family integrases, including VpaIntIA in *V. parahaemolyticus*—that are distinct from the mobile class 1–3 integrons responsible for rapid AMR dissemination in clinical settings [13,14]. Automated bioinformatic tools may misclassify chromosomal super-integron elements as mobile integrons without integrase family typing, leading to overestimation of AMR mobilization risk [15]. Additionally, CALIN elements—chromosomal arrays of integron-like structures retaining *attC* arrays but lacking their own integrase—may serve as repositories for gene cassettes mobilizable by a trans-acting integrase [15,16]. Bacteriophages are similarly important contributors to *Vibrio* genome plasticity and accessory gene diversity [17,18].

In this study, we conducted a six-month environmental surveillance of *V. parahaemolyticus* in coastal seawater and estuarine water at three sites in Haiyan, Jiaxing, Zhejiang, China. By integrating epidemiological monitoring with WGS-based analyses—including population diversity assessment, virulence profiling with BLASTn validation, AMR gene screening, integron characterization with integrase family typing, and prophage prediction—we aimed to characterize this environmental population as an ecological and genomic reservoir with defined public health implications.

## 2. Materials and Methods

### 2.1. Study Sites and Sample Collection

Water samples were collected from three monitoring sites in Haiyan, Jiaxing, Zhejiang, China, from May to October 2024: one coastal seawater site (Site C) and two estuarine sites (Site A and Site B). At each site on each sampling day, six surface-water samples (500 mL each; 0–30 cm depth) were collected using sterile sampling bags from three sub-sampling points spaced approximately 10 m apart, with two replicate samples per point. This design yielded 18 samples per month across three sites and 108 samples over the six-month period. Samples were transported to the laboratory immediately and processed without delay.

### 2.2. Isolation and Identification

Samples were enriched in alkaline peptone water (APW; pH 8.5, 3% NaCl; Hope Bio (Qingdao Haibo Biotechnology Co., Ltd.), Qingdao, China) through two successive steps. For primary enrichment, water samples were mixed with 10× concentrated APW to achieve a final 1× working concentration and incubated at 37 °C for 6–8 h. For secondary enrichment, an aliquot of the primary culture was transferred to fresh 1× APW and incubated at 37 °C for 6–8 h. Enriched cultures were plated on thiosulfate–citrate–bile salts–sucrose (TCBS) agar (Hope Bio (Qingdao Haibo Biotechnology Co., Ltd.), Qingdao, China) and incubated at 37 °C for 18–24 h. Presumptive *V. parahaemolyticus* colonies were subcultured and identified by MALDI-TOF MS (bioMérieux VITEK MS, Marcy-l’Étoile, France) and *tlh* PCR using reagents from MABSKY (Shenzhen Shengkeyuan Biotechnology Co., Ltd., Shenzhen, China). One representative isolate per positive sample was retained for WGS, yielding 39 confirmed isolates.

### 2.3. DNA Extraction and Whole-Genome Sequencing

Genomic DNA was extracted using the VAMNE Magnetic Pathogen DNA/RNA Kit (Vazyme Biotech Co., Ltd., Nanjing, China). WGS was performed on a GeneMind SURFSeq 5000 platform (GeneMind Biosciences Co., Ltd., Shenzhen, China) using paired-end 150 bp sequencing. Raw reads were trimmed with fastp v0.23.1 [19] and assembled with SPAdes v3.15.5 [20] using the --careful option. Assembly statistics were calculated with seqkit v2.7.0 [21], and genome completeness and contamination were assessed with CheckM v1.2.0 [22]. Species identity was confirmed by average nucleotide identity (ANI) using FastANI [23] against RIMD 2210633 (GCF_000196095.1), with ANI ≥ 95% as the species threshold.

### 2.4. Taxonomic Validation of Enlarged-Genome Isolates

Two isolates with notably enlarged genomes (24-09-05 and 24-09-06) were subjected to contig-level taxonomic classification using Kraken2 [24] with the standard database. Classifications were aggregated by taxonomy ID, and total assembly length was apportioned to species-level categories using length-weighted summation. *V. parahaemolyticus* taxonomy IDs (670, 1211705, 1338031–1338034, 1429044) were merged into a single species-level category.

To provide additional context for the taxonomic signal in these two isolates, trimmed reads were mapped to each isolate’s own assembly using BWA-MEM v0.7.18 [25] with default parameters. Per-contig mapping statistics and coverage depth were calculated using samtools v1.18 [26], and a length-weighted mean coverage depth was computed separately for *V. vulnificus*-like contigs and the remaining contigs within each assembly. Per-contig GC content was calculated using seqkit v2.7.0 [21]. Coverage depth distributions and GC content were compared between the two contig groups to assess evidence for a mixed-template signal.

### 2.5. MLST and cgMLST

MLST was performed using the seven-locus *V. parahaemolyticus* scheme (*dnaE*, *gyrB*, *recA*, *dtdS*, *pntA*, *pyrC*, *tnaA*) via PubMLST [27]. Novel allelic profiles were submitted for formal ST assignment. cgMLST was performed using the PubMLST 2254-locus scheme. Pairwise cgMLST allelic distances were calculated from the 2254-locus allelic profiles; for each isolate pair, only loci called in both isolates were compared, and loci missing in either isolate were excluded from that pairwise comparison without being counted as differences. A minimum-spanning tree was constructed from this distance matrix using a custom Python 3 (NumPy) implementation of Prim’s algorithm and displayed with a radial layout (Matplotlib v3.11.1). Zero allelic difference was interpreted as cgMLST-indistinguishable; no allelic-distance threshold was applied for transmission inference.

### 2.6. Virulence Gene Profiling and BLASTn Validation

Virulence genes were screened using ABRicate v1.0.1 with VFDB [28] (identity ≥ 90%, coverage ≥ 80%). Target genes included *tdh*, *trh*, T3SS1-associated loci, T3SS2-associated genes, *tlh*, and *VP1611* (MAM7). For any T3SS1 locus not detected above threshold in specific isolates, confirmatory BLASTn + 2.17.0 searches were performed against the corresponding reference sequence from RIMD 2210633 chromosome 1 (NC_004603.1; megablast; E-value < 1 × 10^−10^). A BLASTn hit with identity ≥ 90% was considered confirmatory; validated loci and affected isolates are reported in Section 3.4.

### 2.7. AMR Gene Profiling

AMR genes were screened with ABRicate v1.0.1 using CARD [29] (identity ≥ 90%, coverage ≥ 80%) and cross-validated with AMRFinderPlus v3.12 [30]. Where gene designations differed between tools, the AMRFinderPlus designation was used as the primary nomenclature. All AMR findings are genotypic predictions only.

### 2.8. Integron Characterization and Integrase Family Typing

Integrons were identified with IntegronFinder v2.0.5 [15,31]. IntegronFinder labels any predicted integrase gene as ‘IntI’ by default, regardless of integrase family; this label does not by itself indicate class 1 mobile integron (IntI1) membership (see integrase family assignment below). Elements were classified structurally as: (i) complete integrons, harboring a predicted integrase gene and at least one *attC* site; (ii) In0-designated elements, harboring a predicted integrase gene but no detectable *attC* sites; or (iii) CALIN elements, retaining *attC* arrays but lacking a predicted integrase gene. For isolates classified as In0-designated (counts and site distribution reported in Section 3.5), full-length integrase proteins were queried by BLASTp against NCBI RefSeq and aligned with the reference VpaIntIA sequence AAK02076.1 from *V. parahaemolyticus* CIP 75.2T [13] using Clustal Omega v1.2.4 [32]. As an independent reproducibility check, integrase proteins from all 39 isolates were also subjected to BLASTp family assignment against a curated reference panel (see Supplementary Table S4 for reference accessions, BLASTp settings, and family-assignment criteria).

### 2.9. Prophage Prediction

Prophages were predicted using geNomad v1.12.0 [33] with default parameters. Elements were retained when they met the prespecified geNomad virus-score (>0.9) and viral-hallmark (≥1) criteria. Terminal-topology categories (Provirus, No terminal repeats, DTR) were recorded for each retained element; Provirus denotes integrated prophages. All three topology categories were collectively retained for downstream length analysis, and their distribution is reported in Section 3.6. Cumulative prophage length per isolate was calculated as the sum of all retained predicted viral-region lengths. Spearman’s rank correlation between cumulative prophage length and total genome size was calculated in R; a sensitivity analysis excluding isolates 24-09-05 and 24-09-06 (identified as taxonomic outliers; see Section 3.2) was also performed.

### 2.10. Environmental Data and Statistical Analysis

Meteorological data for the 30-day period preceding each sampling date—including mean temperature, mean maximum and minimum temperatures, extreme temperatures, total precipitation, and rainy days—were obtained from historical weather records for Haiyan County (Supplementary Table S2). Pearson’s and Spearman’s correlations between mean temperature and total precipitation across the six sampling periods were calculated to evaluate collinearity between these variables (results reported in Section 3.1); given the outcome, they were treated as descriptive contextual variables rather than independent predictors in subsequent analyses.

Culture-based detection proportions were calculated per site and month as the number of positive water samples divided by the number of samples tested. A Cochran–Armitage trend test assessed the monthly trend from June to October, with May excluded because no isolates were detected. Fisher’s exact test with Bonferroni correction was used for pairwise site comparisons. Differences in integron-type prevalence between sites were assessed by Fisher’s exact test. Simpson’s diversity index was calculated from ST frequencies. Because the six within-site monthly samples were collected on the same sampling day and shared the same meteorological context, they were not treated as independent temporal or spatial replicates; all *p*-values were therefore computed at the water-sample level and are interpreted descriptively rather than as inferences to a broader population (see Section 4.7 for further discussion). All analyses were performed in R 4.3.2 [34], with statistical significance defined as *p* < 0.05. The Cochran–Armitage trend result was obtained with the one-sided implementation of stats::prop.trend.test; Fisher’s exact test used stats::fisher.test; and Spearman’s rank correlation used stats::cor.test (method = “spearman”).

## 3. Results

### 3.1. Seasonal and Spatial Occurrence in Relation to Meteorological Context

**Figure 1:**
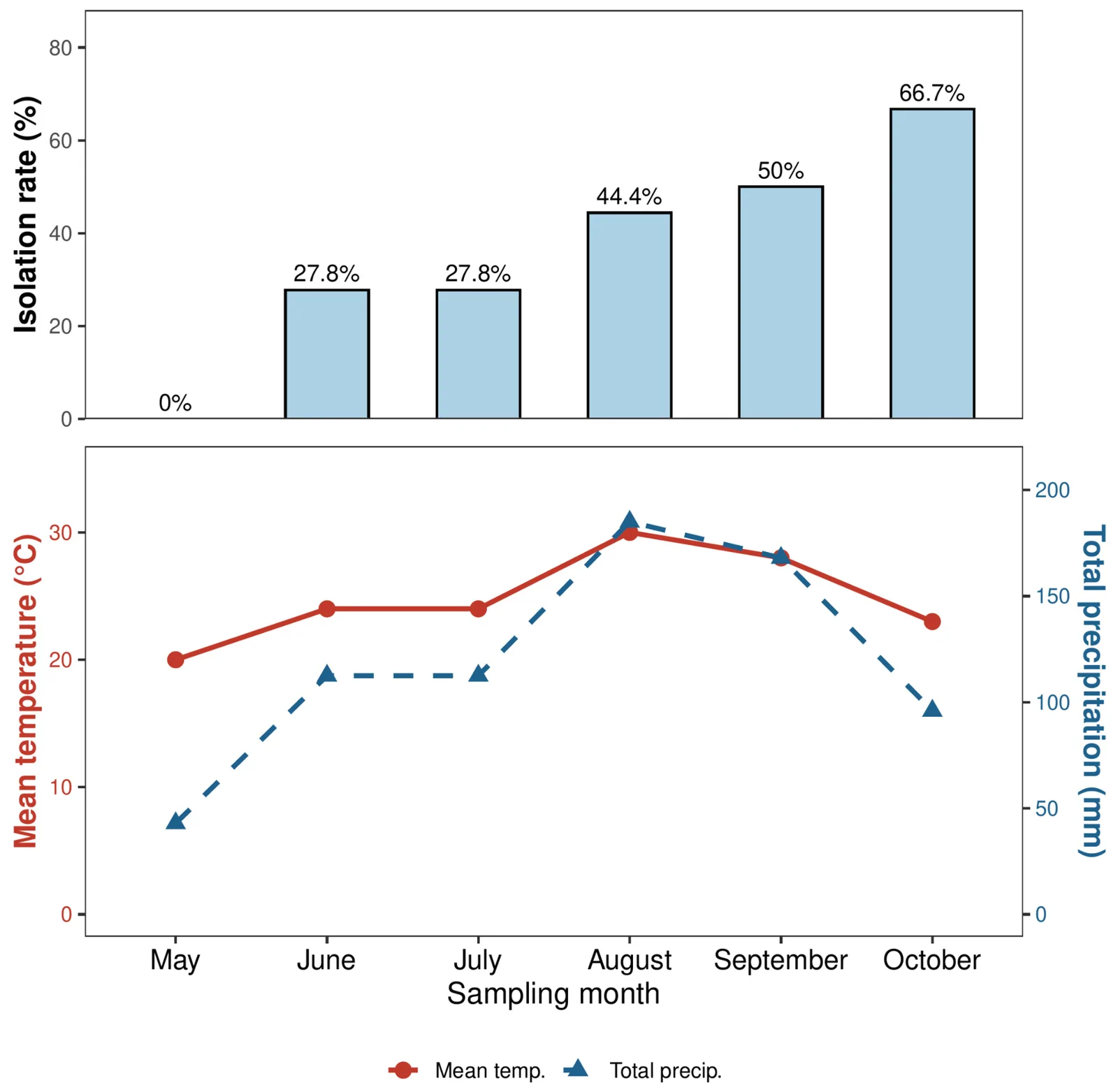
Seasonal occurrence of *V. parahaemolyticus* in relation to meteorological context. Upper panel: overall monthly culture-based detection proportion (all sites combined). Lower panel: preceding 30-day mean air temperature (left *y*-axis) and total precipitation (right *y*-axis).

Among 108 water samples, 39 were culture-positive for *V. parahaemolyticus*, yielding an overall culture-based detection proportion of 36.1% (Table 1). No isolates were recovered in May, when the preceding 30-day mean temperature was 20.0 °C and total precipitation was 43.0 mm—the lowest values recorded during the study period. Detection began in June and the observed culture-based detection proportion increased across the June–October sampling periods (one-sided Cochran–Armitage trend test, *p* = 0.003). This site-month pattern is interpreted descriptively because the within-day subsamples were not independent temporal replicates. This increase coincided with a warm–wet seasonal window in which temperature and precipitation changed together (Supplementary Table S2; Figure 1). Across the six sampling periods, mean temperature and total precipitation were almost perfectly correlated (Pearson’s r = 0.993; Spearman’s ρ = 1.000; *n* = 6; Supplementary Table S2); because temperature and precipitation co-varied substantially, their independent contributions could not be statistically disentangled, and these meteorological variables are presented as seasonal context rather than as independently estimated predictors of detection.

The highest detection proportion was recorded in October (66.7%), despite declining mean temperature (23.0 °C) and reduced precipitation (96.0 mm) relative to August–September. Notably, the September-to-October increase was driven by Site A (0/6 to 3/6), where the three October isolates were cgMLST-indistinguishable (ST4149), whereas Site C had already reached maximal detection from August onward; the October maximum therefore reflects a site-specific event superimposed on saturated coastal detection rather than a synchronous population-wide increase.

Site C showed a higher culture-based detection proportion (77.8%, 28/36) than Site A (8.3%, 3/36) and Site B (22.2%, 8/36) (Fisher’s exact test, *p* < 0.001; sample-level, interpreted descriptively). Pairwise comparisons showed higher detection at Site C than at both Site A *(p* < 0.001) and Site B (*p* < 0.001) after Bonferroni correction; the difference between Site A and Site B was not significant (*p* = 0.189). A temporal lag was observed at the estuarine sites: Site B first yielded positive samples in August, and Site A only in October.

### 3.2. Genome Quality and Taxonomic Validation

All 39 isolates passed WGS quality thresholds (Table 2; Supplementary Table S1). Genome sizes ranged from 4.93 to 6.10 Mb (mean ± SD, 5.24 ± 0.25 Mb). GC content ranged from 44.02% to 45.47% (mean, 45.21%). Contig numbers ranged from 25 to 98 (mean, 45), and N50 values from 207,541 to 577,868 bp (mean, 408,372 bp). CheckM indicated high completeness (97.57–99.85%; mean, 98.65%) and low contamination (0.68–3.73%; mean, 1.54%). ANI confirmed all 39 isolates as *V. parahaemolyticus* (98.18–99.43%; mean, 98.33%).

Two isolates—24-09-05 (ST169; Site C) and 24-09-06 (ST154; Site C)—had notably enlarged genomes (6.10 and 5.98 Mb) and lower GC content (44.02% and 44.05%). Length-weighted Kraken2 analysis assigned approximately 14–15% of their assemblies to *V. vulnificus*, despite ANI confirmation as *V. parahaemolyticus* (Table 3). Read-based mapping provided additional coverage context for the two isolates. The mapping rates were 99.28% for 24-09-05 and 98.94% for 24-09-06; such rates are expected under both single- and mixed-template scenarios and are therefore not discriminatory on their own. Within each assembly, the *V. vulnificus*-like contigs showed length-weighted mean coverage depths of 258.6× and 307.2×, compared with 230.3× and 235.7× for the remaining contigs of the same assemblies, respectively. The coverage distributions were continuous and unimodal, with no separate coverage mode corresponding to the *V. vulnificus*-like contigs. In addition, CheckM contamination estimates for the two isolates were 1.57% and 0.86%, comparable to the dataset mean (~1.5%; Supplementary Table S1), whereas a genuine dual-species mixture contributing approximately 14–15% of an assembly would be expected to duplicate single-copy marker genes and markedly elevate this estimate. The flagged contigs also showed GC contents of 35–40%, below the ~46–47% characteristic of *V. vulnificus* genomes. Together, these results do not support an obvious mixed-template contamination event; they cannot, however, definitively distinguish k-mer misclassification from horizontally acquired DNA or residual mixed-template signal. All contigs from both isolates were assigned to the genus *Vibrio*; no non-*Vibrio* sequences were detected. Genomic features derived from these two isolates were therefore interpreted with caution throughout, and the coverage results were treated as contextual evidence rather than as a definitive explanation of the mixed taxonomic signal.

### 3.3. Population Diversity and Inter-Habitat Exchange

MLST assigned the 39 isolates to 28 distinct STs, including four novel profiles assigned as ST4566 (24-06-04), ST4567 (24-07-02), ST4565 (24-07-05), and ST4396 (24-08-05) (Table 4). No ST was dominant. Simpson’s diversity index was 0.953 overall (Site A: 0.000; Site B: 0.875; Site C: 0.939; Sites A and B combined: 0.860). Because isolate counts differed markedly among sites (*n* = 3, 8, and 28), these values are reported descriptively and are not directly comparable across sites. Reporting Site A and Site B separately reveals that Site A yielded three cgMLST-indistinguishable ST4149 isolates, whereas Site B showed higher observed ST diversity among the recovered isolates. Nearest-neighbour analysis against the *V. parahaemolyticus* PubMLST allele-profile database (queried 20 July 2026) identified ST1679 (3/7 loci differ) as the closest relative of ST4566; ST1317, ST2533, ST3747, and ST4349 (5/7 loci differ) for ST4567; 58 database STs at 5/7 loci difference for ST4565; and 24 database STs at 5/7 loci difference for ST4396. This was an allele-profile comparison; whole-genome comparison with global isolates was not performed because comparable environmental genomes remain limited.

**Figure 2:**
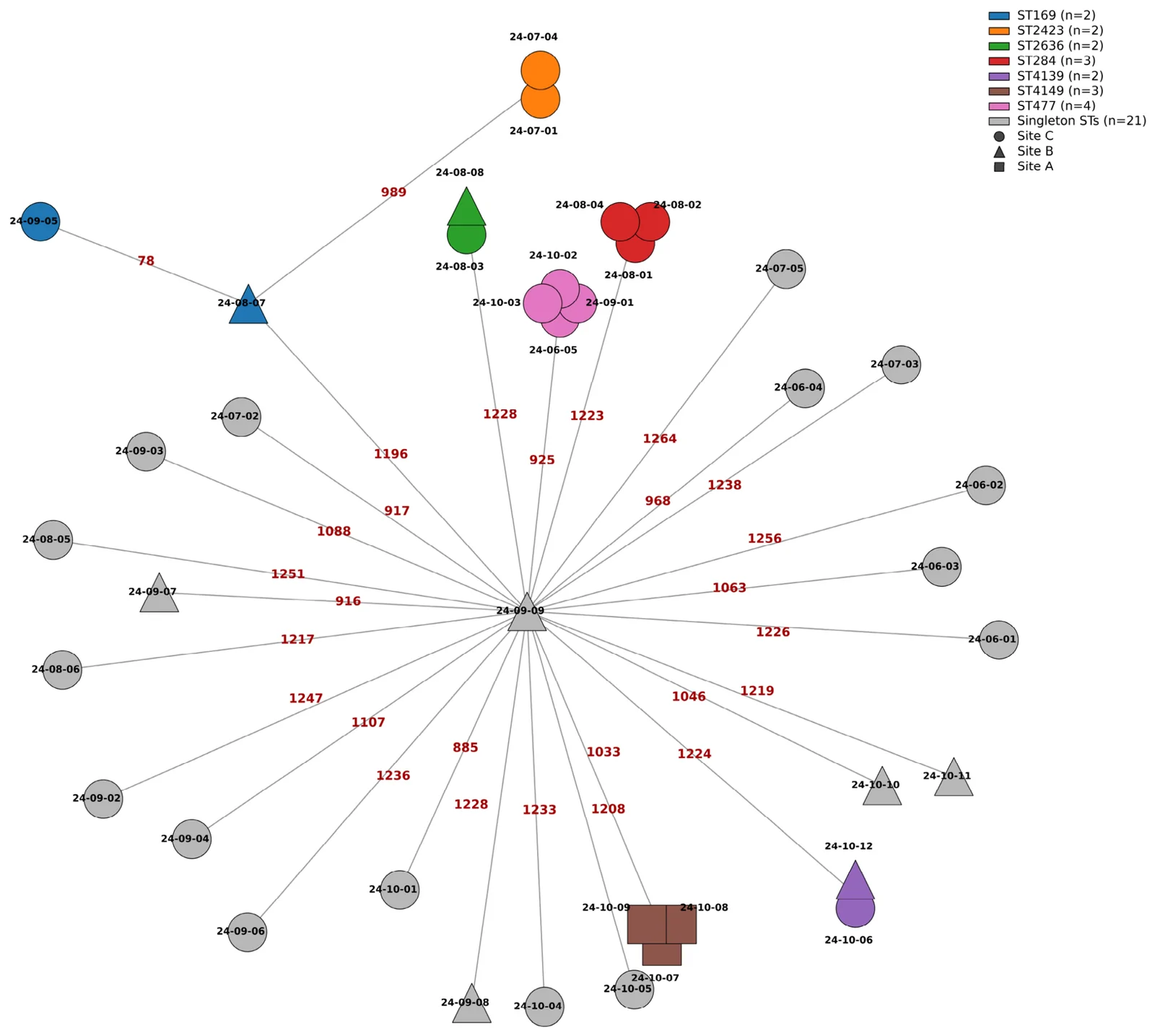
cgMLST-based clustering of 39 environmental *Vibrio parahaemolyticus* isolates. Minimum-spanning tree based on pairwise allelic distances from the 2254-locus scheme. Edge labels indicate pairwise allelic distances on non-zero-distance branches (smallest non-zero distance: 78 alleles). The layout is descriptive and does not represent phylogenetic ancestry or transmission direction.

The 2254-locus cgMLST distance matrix showed multiple lineages without a dominant zero-distance group (Figure 2). Zero allelic difference was interpreted as cgMLST-indistinguishable; non-zero distances were not assigned a transmission threshold. Six zero-distance cgMLST groups were identified, encompassing 16 isolates: ST477 (*n* = 4), ST284 (*n* = 3), ST4149 (*n* = 3), ST2423 (*n* = 2), ST2636 (*n* = 2), and ST4139 (*n* = 2), and all zero-distance pairs shared at least 1636 callable loci. The three ST4149 isolates from Site A in October shared an identical VpaIntIA sequence, with concordant prophage profiles. The three ST284 isolates from Site C in August also showed zero allelic difference. These concordant observations are compatible with local clonality and are consistent with a single introduction event. Four singleton STs (ST4110, ST4111, ST4112, ST4113) were recovered in September—three from Site C and one (24-09-07, ST4113) from Site B; the smallest pairwise cgMLST distance among them was 1186 alleles, so they are reported as a descriptive September occurrence pattern; the large pairwise distances indicate these four STs are distinct lineages rather than a tight cgMLST cluster.

Three STs were shared between Site C and estuarine sites: ST2636 (Site C and Site B, August), ST169 (Site B in August, Site C in September), and ST4139 (Site C and Site B, October). The ST2636 and ST4139 pairs were cgMLST-indistinguishable across sites (zero allelic differences), providing strain-level evidence of inter-habitat exchange; the two ST169 isolates differed by 78 alleles—the smallest non-zero pairwise distance in the dataset—and therefore support only ST-level, not strain-level, sharing. ST169 was detected earlier at Site B than at Site C, indicating that occurrence was not strictly unidirectional.

### 3.4. Virulence Profile

All 39 isolates lacked *tdh*, *trh*, and all screened T3SS2 components (0/39). *tlh* and *VP1611* (MAM7) were detected in all isolates (100%) (Table 5).

Initial ABRicate/VFDB screening detected 36 of 39 T3SS1 loci in all isolates. Three loci—*vcrV*, *vopB*, and *vscP*—were below the detection threshold in specific isolates. BLASTn validation confirmed all three present in the corresponding assemblies (identity ≥ 96.76%; E-value = 0; Table 6). Reduced query coverage (84–94%) was compatible with strain-specific allelic divergence of approximately 3% (2.8–3.2%) rather than true gene absence. After validation, a complete 39-locus T3SS1 repertoire was confirmed in all 39 isolates. Query coverage was calculated relative to the full-length reference ORF and is reported in Table 6.

### 3.5. AMR Gene Prevalence and Integron/VpaIntIA Characterization

All 39 isolates carried an intrinsic CARB-type β-lactamase: CARB-21 (21/39, 53.8%) or CARB-23 (18/39, 46.2%). These were mutually exclusive and represent constitutive chromosomal features (Supplementary Table S1). Isolate 24-06-02 additionally carried *sul2*. One isolate, 24-09-07 (ST4113; Site B; September), carried eight predicted AMR genes spanning seven resistance classes (Table 7); however, phenotypic susceptibility was not confirmed.

IntegronFinder identified complete integrons in 33/39 isolates (84.6%), In0-designated elements in 6/39 (15.4%), and CALIN elements in all 39 isolates (100%; range, 1–24; mean, 8.9) (Table 8; Supplementary Table S1). In0-designated elements showed an uneven site distribution (Fisher’s exact test, *p* = 0.003; sample-level): all three Site A isolates harbored In0 elements (100%), versus 3/28 at Site C (10.7%) and 0/8 at Site B; because the three Site A isolates were cgMLST-indistinguishable, this distribution is interpreted descriptively.

BLASTp family assignment of integrases across all 39 isolates identified VpaIntIA as the best match in all 39 cases, with identities ranging from 95.9% to 100.0% (mean, 99.2%). The six In0-associated integrases were therefore interpreted as VpaIntIA-type chromosomal super-integron integrases rather than mobile class 1–3 integron integrases (Table 9; Supplementary Tables S3 and S4). Four VpaIntIA amino-acid variants were identified among the six In0-designated isolates. The three ST4149 isolates shared an identical sequence (Variant A), consistent with their cgMLST-indistinguishable relationship.

Variant D (R192Q; 4 aa differences) includes a non-conservative Arg → Gln substitution outside the catalytic-site residues evaluated by sequence alignment with characterized tyrosine recombinases. This substitution is therefore not located at a known catalytic residue; however, sequence comparison alone cannot exclude effects on protein folding, DNA-binding, or recombination efficiency, and its functional impact on integrase activity remains untested.

Isolate 24-09-07 harbored a dual-element architecture: a complete integron on contig_5 containing a predicted integrase gene (BLASTp-confirmed as *VpaIntIA*; Table 9), six *attC* sites, and cassette ORFs without known resistance genes; and a CALIN element on contig_35 in which *dfrA31* (designated *dfrA6* by CARD; see Table 7) and *qnrVC5* were co-localized with *attC* sites, a genomic context compatible with possible mobilization in trans; mobilization was not experimentally demonstrated.

### 3.6. Prophage Content

**Figure 3:**
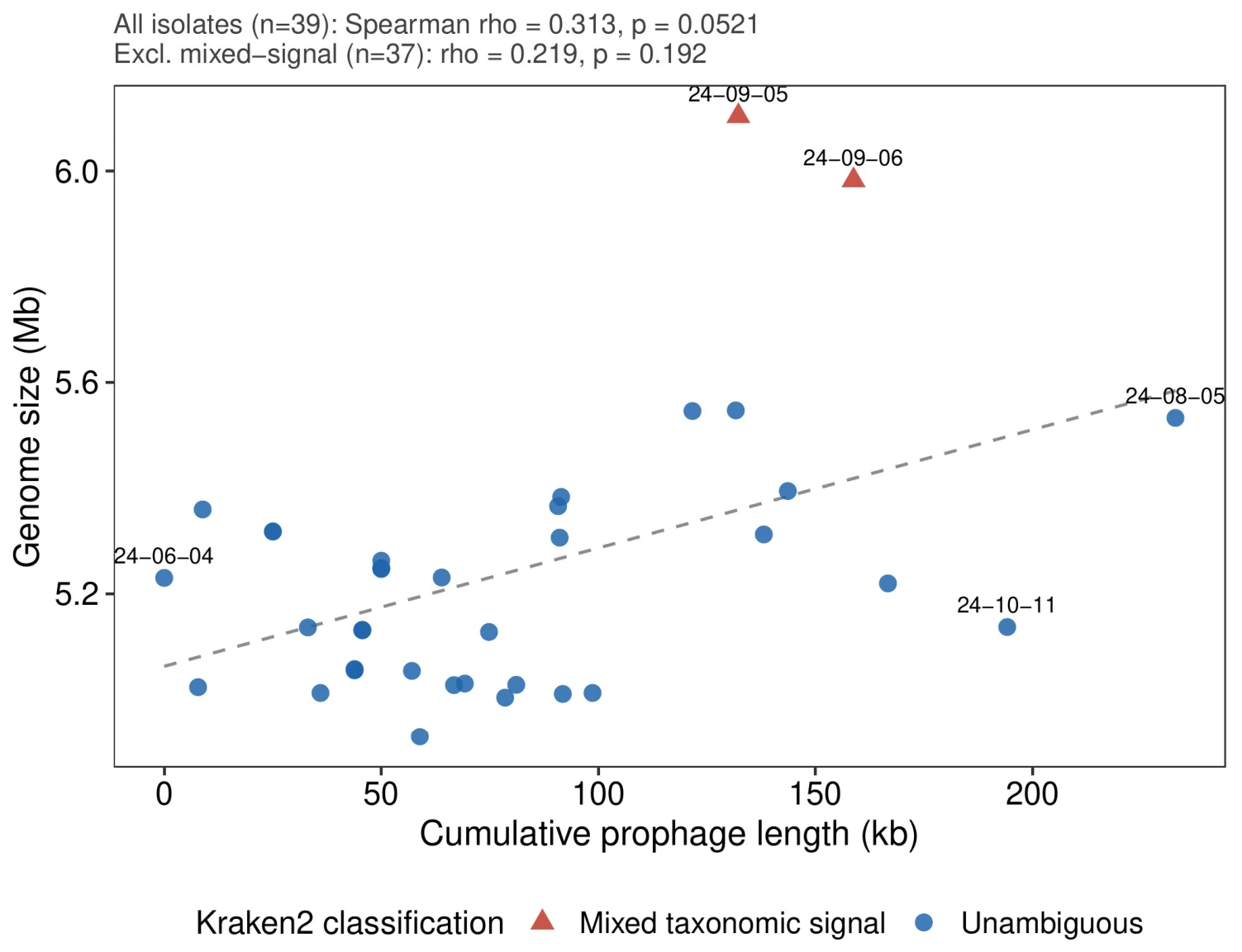
Cumulative retained prophage length versus genome size across 39 isolates. Red triangles indicate isolates 24-09-05 and 24-09-06 with mixed *Vibrio* taxonomic signals. Spearman’s *ρ* = 0.313, *p* = 0.052 (*n* = 39); *ρ* = 0.219, *p* = 0.192 (*n* = 37, excluding mixed-signal isolates).

A total of 116 retained prophages were identified with geNomad, distributed across 38/39 isolates (97.4%). The sole prophage-free isolate was 24-06-04 (ST4566; Site C; June). Filamentous phage-related annotations were detected in 20/38 prophage-positive isolates (52.6%). Cumulative retained prophage length ranged from 0 to 232.9 kb (Supplementary Table S1); excluding the two mixed-signal isolates (24-09-05 and 24-09-06), the mean ± SD was 74.2 ± 51.3 kb. Across all 39 isolates, cumulative prophage length was not significantly correlated with genome size (Spearman’s *ρ* = 0.313, *p* = 0.052; Figure 3). Sensitivity analysis excluding the two mixed-signal isolates yielded an even weaker correlation (*ρ* = 0.219, *p* = 0.192; *n* = 37). Concordant prophage profiles were observed among the ST284, ST2636, and ST4149 groups; these profiles provide supportive comparative evidence but do not constitute experimental validation of viral activity or completeness.

Extended characterization of the 116 retained regions showed topology categories of Provirus for 95/116 (81.9%), no terminal repeats for 16/116 (13.8%), and DTR for 5/116 (4.3%) (here “Provirus” denotes the geNomad topological category, not formal element completeness); virus scores ranged from 0.905 to 0.999 (median 0.995) with 1–17 viral hallmark genes (median 3). Functional annotation covered 1049 genes with a non-empty annotation description, of which 563 were virus-hallmark genes. By functional category, 680 of the 1049 genes were assigned to structural modules (capsid/tail/portal; *n* = 309), replication/packaging (*n* = 174), transcriptional regulation (*n* = 131), integration/excision (*n* = 52), or lysis functions (*n* = 14); the remaining 369 genes carried other annotations. Taxonomic assignment was dominated by Caudoviricetes (class; 70/116, 60.3%), followed by Inoviridae (family; 23/116, 19.8%), Tectiliviricetes (class; 16/116, 13.8%), Corticoviridae (family; 4/116, 3.4%), and Faserviricetes (class; 3/116, 2.6%). These geNomad-derived metrics are not equivalent to a formal completeness assessment.

## 4. Discussion

### 4.1. Environmental Occurrence Reflects a Warm–Wet Seasonal Window and Coastal–Estuarine Heterogeneity

This study observed an increase in the culture-based detection proportion during a warm–wet seasonal window, with detection rising from 27.8% in June to 66.7% in October. The absence of detection in May is consistent with lower thermal suitability during the preceding 30-day period, when the mean temperature was 20 °C and the extreme minimum reached 7 °C. Detection was observed in June–July when the 30-day mean air temperature was 24 °C; this observation does not establish a temperature threshold. Classical ecological work in Chesapeake Bay demonstrated that *V. parahaemolyticus* abundance increases when water temperatures exceed approximately 15–20 °C [7], and subsequent studies confirmed temperature as a primary driver of seasonal occurrence in coastal waters and seafood [6,9]. The higher apparent threshold observed here (~24 °C mean air temperature) likely reflects the use of air temperature as a proxy for water temperature and the enrichment-based detection approach, rather than a fundamentally different thermal ecology.

However, the October peak cannot be explained by temperature alone. Mean temperature declined from 30 °C in August to 23 °C in October, yet the overall culture-based detection proportion reached its highest level in October. This pattern suggests that *V. parahaemolyticus* abundance in this setting is shaped not simply by instantaneous air temperature but by a combination of thermal suitability, hydrological conditions, and potential lag effects. The 30-day cumulative precipitation was highest in August and September (185.0 and 168.0 mm), preceding the October isolation peak. Previous studies have shown that plankton composition, nutrient availability, and hydrological inputs can modulate *Vibrio* seasonality beyond temperature alone [9]. In the Haiyan coastal system, rainfall-driven runoff may increase nutrient and organic matter inputs into coastal waters, indirectly supporting heterotrophic bacterial growth. At the same time, seawater thermal inertia may allow coastal water temperatures to remain favorable after air temperature declines. Because only meteorological rather than in situ water-quality data were available, this temperature–precipitation interpretation should be regarded as a plausible ecological explanation rather than a formally tested mechanism.

The strong spatial contrast between the coastal site and the estuarine sites further supports habitat-specific environmental filtering. Site C showed sustained high detection from June onward, whereas the estuarine sites showed delayed detection. This pattern is consistent with the halophilic nature of *V. parahaemolyticus*: coastal seawater likely provides more stable salinity, whereas estuarine habitats are more strongly affected by freshwater pulses during heavy rainfall periods.

Importantly, the data do not support a strictly unidirectional coastal-to-estuarine dispersal model. Shared STs between Site C and Site B indicate inter-habitat exchange; in particular, the cgMLST-indistinguishable ST2636 and ST4139 pairs provide strain-level evidence, whereas the two ST169 isolates (Site B in August, Site C in September) differed by 78 alleles. This temporal pattern is compatible with bidirectional or non-unidirectional occurrence across the sampled habitats, but the sampling design does not establish reservoir function or the direction of dispersal.

### 4.2. A Highly Diverse Population Structure Argues Against Single-Clone Expansion

The seasonal increase in culture-based detection proportion was not driven by expansion of a single dominant clone. Instead, the population showed high genetic diversity, with 28 STs among 39 isolates and a Simpson diversity index of 0.953. The cgMLST distance pattern was consistent with multiple lineages and no dominant zero-distance group. Thus, the observed seasonal increase is more consistent with a multi-lineage detection pattern than with expansion of a single clone, although the sampling design cannot directly measure reservoir activation or proliferation.

Within this diverse background, several localized clonal clusters were detected. The three ST284 isolates from Site C in August and the three ST4149 isolates from Site A in October were cgMLST-indistinguishable groups. By contrast, the four singleton STs ST4110–ST4113 did not form a tight cgMLST cluster (smallest pairwise distance among them, 1186 alleles); they are therefore reported as a descriptive September pattern rather than a short-term local expansion event. The data are compatible with a diverse environmental population containing occasional same-ST or cgMLST-indistinguishable groups, but they are consistent with a “diverse reservoir plus localized pulse expansion” model, proposed as a working hypothesis pending longer-term sampling.

The detection of four novel STs within a six-month survey also highlights the under-sampled diversity of environmental *V. parahaemolyticus* in local coastal ecosystems. Such diversity is epidemiologically relevant because environmental populations provide the genomic background from which clinically relevant lineages may emerge through acquisition of virulence or resistance determinants.

### 4.3. Virulence Profile: Non-Pandemic Does Not Mean Biologically Irrelevant

All isolates lacked *tdh*, *trh*, and T3SS2, indicating that this environmental population is distinct from pandemic clinical *V. parahaemolyticus* lineages. This finding suggests that the immediate risk of classical hemolytic gastroenteritis associated with these isolates is low. However, absence of pandemic markers should not be interpreted as ecological or public-health irrelevance.

All 39 isolates retained *tlh*, MAM7, and a complete T3SS1 repertoire. T3SS1 is broadly conserved in *V. parahaemolyticus* and contributes to cytotoxicity, interactions with eukaryotic cells, and environmental fitness [3,4]. MAM7 is involved in adhesion to host cells and abiotic surfaces, potentially supporting biofilm formation and persistence in marine environments [5]. Therefore, the virulence profile observed here is best described as a conserved environmental virulence scaffold: not characteristic of pandemic clinical strains, but still relevant to environmental survival, host association, and future evolutionary potential.

The BLASTn verification of *vcrV*, *vopB*, and *vscP* also has a methodological implication. These loci were initially missed by ABRicate/VFDB in specific isolates but were confirmed present by direct BLASTn. Their identity remained high (96.76–97.23%), while query coverage was reduced (84–94%), a pattern compatible with approximately 3% strain-specific allelic divergence (2.8–3.2%) rather than true absence. This result illustrates a general challenge in environmental genomics: databases built primarily from clinical strains may under-detect divergent alleles in environmental isolates when strict identity and coverage thresholds are applied.

### 4.4. AMR and Integron Findings Identify Latent Mobilome-Associated Risk

The overall AMR burden was low. Most isolates carried only intrinsic CARB-type β-lactamases. Only two isolates carried acquired AMR determinants beyond CARB: 24-06-02 with *sul2* and 24-09-07 with a multidrug-resistant genotype. This low prevalence is reassuring from an immediate resistance-surveillance perspective.

Nevertheless, isolate 24-09-07 is important because of its genomic context. This isolate was recovered from Site B, an estuarine site, and carried eight AMR genes spanning seven resistance classes, including *dfrA31* and *qnrVC5*. The co-localization of these two resistance genes within a CALIN element suggests cassette-associated resistance storage in an integron-derived structure. Although the physical linkage between the complete integron on contig_5 and the CALIN on contig_35 cannot be fully resolved with short-read data, the presence of a predicted integrase elsewhere in the same genome supports the possibility of cassette mobilization in trans.

The recovery of this single predicted-MDR isolate at an estuarine site is a single observation; the study design cannot determine why it occurred at that site, but it is consistent with concerns raised in previous studies regarding estuarine interfaces. Estuaries and coastal receiving waters are increasingly recognized as convergence zones for antibiotic residues, resistant bacteria, and mobile resistance elements transported from wastewater, agricultural runoff, aquaculture systems, and other terrestrial sources [35,36]. Aquatic systems can maintain, mix, and mobilize AMR determinants across environmental and anthropogenic bacterial communities [37]. The presence of *qnrVC5* and *dfrA31* in this isolate is compatible with concerns raised in previous studies regarding estuarine interfaces [36,38], but the present dataset does not permit inference of greater AMR acquisition at estuarine than at coastal sites. Although only one predicted-MDR isolate was detected, its recovery supports continued inclusion of estuarine sites in environmental *Vibrio* AMR surveillance without implying an estuary-specific causal mechanism.

The integron analysis provides one of the most important findings of this study. IntegronFinder identified In0-designated elements in six isolates, and BLASTp assignment identified VpaIntIA as the best integrase match across all 39 isolates (95.9–100.0% identity; mean, 99.2%). The six In0-associated elements were therefore interpreted as VpaIntIA-type chromosomal super-integron structures rather than mobile class 1–3 integrons. This distinction is critical. Chromosomal super-integrons were first characterized as ancient cassette-capture systems in *Vibrio* and related bacteria [13] and are now recognized as long-term platforms for genome evolution and environmental adaptation rather than immediate vehicles of clinical AMR dissemination [11,14,16]. In *V. parahaemolyticus*, the chromosomal super-integron contains numerous gene cassettes, many of which remain functionally uncharacterized [4,13]. Our finding that all six In0-designated elements were VpaIntIA variants therefore reinforces the methodological argument that automated integron detection in *Vibrio* genomes must be coupled with integrase family typing to avoid conflating ancient chromosomal architecture with recent mobile AMR vectors. Without such typing, environmental surveillance studies risk overestimating the prevalence of mobile resistance integrons [14,15].

Together, the CALIN-associated resistance genes in 24-09-07, universal CALIN carriage, and VpaIntIA-type super-integron architecture indicate that cassette-associated genomic structures are present in this population, although acquired AMR genes were uncommon and actual horizontal transfer or mobilization was not assessed. This is the main public-health implication of the mobilome results: the present population is not highly resistant as a whole, but it contains structural components that could support future AMR acquisition under appropriate selective pressures.

### 4.5. Prophages Contribute to Accessory Diversity but Do Not Explain Genome Size Variation

Retained prophages were detected in 38 of 39 isolates, with 116 regions identified across the dataset. Prophages are major contributors to bacterial genome evolution and frequently mediate acquisition of accessory traits, including virulence-associated genes in *Vibrio* species [17,18]. Filamentous phage-related annotations were observed in a subset of isolates; these computational annotations do not establish active phage replication or chronic phage–host interactions.

However, prophage content should not be overinterpreted as the main driver of genome size variation. Cumulative prophage length showed only a weak, non-significant association with genome size across all isolates (Spearman’s *ρ* = 0.313, *p* = 0.052), and the association further weakened after excluding the two isolates with mixed *Vibrio* taxonomic signals (*ρ* = 0.219, *p* = 0.192). Moreover, the isolates with the highest prophage loads were not the isolates with the largest genomes. Thus, the retained prophage annotations were associated with accessory genome content in this dataset, but their quantitative contribution to genome size was not supported by the observed correlation.

The two largest genomes, 24-09-05 and 24-09-06, require cautious interpretation. Both were confirmed as *V. parahaemolyticus* by ANI, but length-weighted Kraken2 analysis indicated that approximately 14–15% of their assemblies were assigned to *V. vulnificus*. Therefore, genome expansion in these two isolates should not be attributed to putative viral region accumulation without long-read or read-based validation.

### 4.6. Implications for Environmental Genomic Surveillance

The findings of this study support a surveillance model in which environmental *V. parahaemolyticus* is evaluated not only by the presence of classical virulence genes but also by population diversity and mobilome-associated genomic features. In Haiyan, the isolates lacked pandemic virulence markers, but they showed high observed population diversity, complete T3SS1, CALIN annotations in all isolates, VpaIntIA-type integrase matches, widespread prophages, and one estuarine isolate carrying a predicted MDR gene profile with CALIN-associated loci.

This combination suggests limited immediate pandemic-like virulence but measurable evolutionary and public-health relevance. Future surveillance should therefore integrate: (i) environmental occurrence data, including temperature and rainfall context; (ii) population genomic diversity; (iii) virulence marker screening with validation of divergent alleles; (iv) AMR gene detection; and (v) integrase family typing to distinguish VpaIntIA-type chromosomal super-integron integrases from mobile resistance-associated integrases. Such an integrated framework is especially important at coastal–estuarine interfaces where environmental, animal, aquaculture, and human-associated bacterial gene pools may overlap [35,36,37,38].

Based on the workflow applied in this study, we further recommend a four-step operational protocol for incorporating integrase family typing into standardized *Vibrio* surveillance: (1) routine structural calling with IntegronFinder on every WGS assembly; (2) integrase family verification via BLASTp against a curated reference panel (VpaIntIA, IntI1, IntI2, IntI3, VchIntIA; parameters detailed in Supplementary Table S4); (3) separate reporting of integrase-bearing element counts, family assignment, and resistance-gene linkage; and (4) maintenance of a versioned reference database for cross-laboratory reproducibility.

### 4.7. Limitations

Several limitations should be acknowledged. First, sampling was conducted monthly at three sites over six months. Although this design captured clear seasonal and spatial patterns, it cannot fully resolve short-term dynamics following rainfall, tidal events, or temperature fluctuations. More frequent sampling across multiple years would be required to assess interannual stability.

Second, the six replicate samples collected at each site per month were obtained on a single sampling day from nearby sub-sampling points. These replicates improve local detection sensitivity but are not fully independent temporal observations. Therefore, the reported site-month comparisons should be interpreted as descriptive surveillance inferences rather than as estimates based on six independent temporal replicates.

Third, meteorological data were based on historical air-temperature and precipitation records rather than direct in situ measurements. Water temperature, salinity, turbidity, dissolved oxygen, nutrients, chlorophyll a, and pH were not measured. As a result, the proposed roles of rainfall-driven runoff, salinity shifts, and nutrient enrichment remain ecological interpretations rather than directly tested mechanisms.

Fourth, only one isolate per positive water sample was sequenced, which may underestimate within-sample strain diversity.

Fifth, AMR findings are genotypic predictions only. Phenotypic antimicrobial susceptibility testing was not performed.

Sixth, short-read assemblies limit structural resolution of complex genomic regions, including the split integron/CALIN architecture in 24-09-07 and the enlarged genomes of 24-09-05 and 24-09-06. Long-read sequencing would be valuable for definitive structural resolution.

Seventh, the mixed *Vibrio* taxonomic signals in 24-09-05 and 24-09-06 require cautious interpretation. Read-based mapping showed high overall mapping rates and continuous, unimodal coverage distributions for the *V. vulnificus*-like contigs, and CheckM contamination estimates were low (1.57% and 0.86%); however, these results did not resolve whether the signal reflected k-mer misclassification, horizontally acquired DNA, or residual mixed-template signal. Long-read sequencing and additional targeted validation would be needed for definitive structural and taxonomic resolution.

Eighth, expression and cytotoxicity of T3SS1 were not experimentally assessed.

Ninth, prophage characterization relied on computational prediction alone: a formal CheckV-based completeness assessment was not performed, prophage induction was not experimentally validated, functional annotation of predicted prophage genes depended on sequence-homology inference without experimental verification of gene function, and taxonomic assignment of prophage regions was based on geNomad topology and marker-gene scores, which may not resolve the true host range or ancestry of integrated elements. These limitations mean that the prophage catalogue reported here should be interpreted as a structural and compositional overview rather than a functionally or taxonomically validated characterization.

Tenth, the double-enrichment procedure introduces selection bias favouring cells that survive and proliferate under the specific enrichment and plating conditions; the recovered isolates therefore represent the cultivable, enrichment-dominated fraction rather than the full community.

Despite these limitations, the central conclusions—seasonal and spatial structuring, high population diversity, absence of pandemic virulence markers, conservation of T3SS1/MAM7, low overall acquired AMR burden, and the presence of VpaIntIA-type super-integron architecture—are supported by multiple independent genomic analyses.

## 5. Conclusions

This study provides a focused genomic surveillance analysis of environmental *Vibrio* parahaemolyticus from coastal and estuarine waters of Haiyan, Zhejiang, China. Culture-based detection proportions increased across the sampled warm–wet seasonal window and were higher at the coastal seawater site than at the estuarine sites. These observations are consistent with habitat-associated spatial structuring and episodic inter-habitat occurrence, but do not establish environmental filtering or dispersal direction.

The population was highly diverse, with 28 STs among 39 isolates and no dominant clone, indicating that the observed seasonal increase involved multiple detected lineages rather than a single dominant lineage; the sampling design does not directly establish environmental proliferation. All isolates lacked *tdh*, *trh*, and T3SS2, but retained complete T3SS1 and MAM7, defining a non-pandemic but conserved environmental virulence scaffold.

Acquired AMR genes were uncommon, but one estuarine isolate carried a predicted MDR gene profile including CALIN-associated *dfrA31* and *qnrVC5*. BLASTp family typing identified VpaIntIA as the best match for integrases across all 39 isolates (95.9–100.0% identity; mean, 99.2%); the six In0-associated elements were therefore interpreted as VpaIntIA-type chromosomal super-integron structures rather than mobile class 1–3 integrons. This is a key methodological implication of the study: accurate interpretation of integron predictions in environmental *Vibrio* genomes requires integrase family typing. Based on this workflow, we recommend that integrase family typing be incorporated into routine environmental *Vibrio* genomic surveillance.

Prophages were widespread in the geNomad predictions, and their cumulative length was not significantly correlated with genome size. Overall, Haiyan coastal waters harbor a genetically diverse and genomically plastic environmental *V. parahaemolyticus* reservoir with limited immediate pandemic-like virulence but measurable mobilome-associated AMR potential, particularly at the coastal–estuarine interface.

## Figures and Tables

**Table 1:** Monthly *V. parahaemolyticus* culture-based detection proportions by sampling site.

Site	Water Type	May	June	July	August	September	October	Total
Site C	Coastal seawater	0/6 (0%)	5/6 (83.3%)	5/6 (83.3%)	6/6 (100%)	6/6 (100%)	6/6 (100%)	28/36 (77.8%)
Site A	Estuary A	0/6 (0%)	0/6 (0%)	0/6 (0%)	0/6 (0%)	0/6 (0%)	3/6 (50.0%)	3/36 (8.3%)
Site B	Estuary B	0/6 (0%)	0/6 (0%)	0/6 (0%)	2/6 (33.3%)	3/6 (50.0%)	3/6 (50.0%)	8/36 (22.2%)
Total	—	0/18 (0%)	5/18 (27.8%)	5/18 (27.8%)	8/18 (44.4%)	9/18 (50.0%)	12/18 (66.7%)	39/108 (36.1%)

Values indicate culture-positive samples/total samples (%). Site C, coastal seawater; Site A and Site B, estuarine waters. Cochran–Armitage trend test from June to October: *p* = 0.003. Fisher’s exact test with Bonferroni correction: Site C vs. Site A and Site C vs. Site B, *p* < 0.001; Site A vs. Site B, *p* = 0.189. Percentages represent the proportion of water samples positive for *V. parahaemolyticus* per site per month, not the proportion of isolates.

**Table 2:** Genome assembly statistics for 39 *V. parahaemolyticus* isolates.

Parameter	Mean ± SD	Minimum (Isolate)	Maximum (Isolate)
Genome size (Mb)	5.24 ± 0.25	4.93 (24-09-09)	6.10 (24-09-05)
GC content (%)	45.21 ± 0.30	44.02 (24-09-05)	45.47 (24-06-02)
Contigs (n)	45 ± 16	25 (24-10-11)	98 (24-08-08)
N50 (bp)	408,372	207,541 (24-08-08)	577,868 (24-10-11)
Completeness (%)	98.65 ± 0.29	97.57 (24-09-02)	99.85 (24-06-03)
Contamination (%)	1.54 ± 0.67	0.68 (24-08-06)	3.73 (24-06-02)
ANI vs. RIMD 2210633 (%)	98.33 ± 0.19	98.18 (24-10-01)	99.43 (24-10-05)

**Table 3:** Kraken2 length-weighted taxonomic classification of enlarged-genome isolates.

Isolate	Total Assembly (Mb)	*V. parahaemolyticus* (%)	*V. vulnificus* (%)	Other *Vibrio* (%)
24-09-05	6.10	85.4	14.5	0.1
24-09-06	5.98	84.8	14.6	0.6

All contigs were assigned to genus *Vibrio*; no non-*Vibrio* sequences were detected.

**Table 4:** MLST sequence type distribution among 39 isolates.

ST	*n*	Site(s)	Month(s)	Notes
ST477	4	Site C	Jun, Sep, Oct	Most frequent
ST284	3	Site C	Aug	Tight cgMLST cluster
ST4149	3	Site A	Oct	cgMLST-indistinguishable; consistent with single introduction
ST2423	2	Site C	Jul	—
ST2636	2	Site C, Site B	Aug	Inter-habitat shared
ST169	2	Site B, Site C	Aug, Sep	Earlier at estuarine site
ST4139	2	Site C, Site B	Oct	Inter-habitat shared
ST4113	1	Site B	Sep	Predicted MDR gene profile
ST1856	1	Site C	Jun	Acquired *sul2*
ST154	1	Site C	Sep	Enlarged genome
ST890	1	Site B	Oct	High prophage load
ST4566 ^a^	1	Site C	Jun	Novel ST; prophage-free
ST4567 ^a^	1	Site C	Jul	Novel ST
ST4565 ^a^	1	Site C	Jul	Novel ST
ST4396 ^a^	1	Site C	Aug	Novel ST; High prophage load
Remaining 13 STs	1 each	Mixed	Mixed	Singleton STs
Total	39	—	—	28 distinct STs

^a^ Novel allelic profiles formally assigned by PubMLST.

**Table 5:** Summary virulence gene detection across 39 isolates.

Virulence Gene/System	Function	Detected/39	%	Method
*tdh*	Thermostable direct hemolysin	0/39	0	ABRicate/VFDB
*trh*	TDH-related hemolysin	0/39	0	ABRicate/VFDB
T3SS2 genes	Enterotoxic T3SS	0/39	0	ABRicate/VFDB
*tlh*	Thermolabile hemolysin	39/39	100	ABRicate/VFDB
*VP1611* (MAM7)	Multivalent adhesion molecule	39/39	100	ABRicate/VFDB
T3SS1 repertoire	Regulation, apparatus, effectors	39/39	100	ABRicate/VFDB
*vcrV* ^a^	Needle tip/translocon platform	39/39	100	ABRicate + BLASTn
*vopB* ^a^	Translocator pore protein	39/39	100	ABRicate + BLASTn
*vscP* ^a^	Needle length control	39/39	100	ABRicate + BLASTn

^a^ Confirmed by BLASTn after initial below-threshold detection (see Table 6).

**Table 6:** BLASTn validation of T3SS1 loci initially below ABRicate threshold.

Gene	Locus Tag	Isolate(s)	Identity (%)	Query Coverage (%)	E-Value	Conclusion
*vcrV*	VP1659	24-08-03, 24-08-08	96.98	84	0	Present; allelic variant
*vopB*	VP1657	24-06-02	97.23	94	0	Present; allelic variant
*vscP*	VP1670	24-09-07	96.76	91	0	Present; allelic variant

Reference sequences from RIMD 2210633 chromosome 1 (NC_004603.1).

**Table 7:** Predicted AMR genes in isolate 24-09-07 (ST4113; Site B; September).

Gene	Contig	Resistance Class	Identity (%)	Tool	Mechanism	Genomic Context
CARB-21	contig_6	β-lactam	98.29	CARD	β-lactamase	Intrinsic chromosomal
*tet*(59)	contig_30	Tetracycline	99.67	CARD	Efflux pump	Independent locus
*APH*(6)-Id	contig_31	Aminoglycoside	99.88	CARD	Phosphotransferase	Composite transposon-like
*APH*(3″)-Ib	contig_31	Aminoglycoside	99.75	CARD	Phosphotransferase	Composite transposon-like
*sul2*	contig_31	Sulfonamide	100.00	CARD	Target replacement	Composite transposon-like
*dfrA6* ^a^	contig_35	Diaminopyrimidine	96.41	CARD	Target replacement	CALIN/*attC* array
*qnrVC5*	contig_35	Fluoroquinolone	100.00	CARD	Target protection	CALIN/*attC* array
*floR*	contig_36	Phenicol	98.03	CARD	Efflux pump	Independent locus

^a^ Detected by CARD as *dfrA6* (96.41% identity). AMRFinderPlus v3.12 designated the same locus as *dfrA31* (100% identity). The AMRFinderPlus designation was used as the primary nomenclature in the main text. Findings are genotypic predictions only.

**Table 8:** Integron and CALIN element distribution by site.

Site	Isolates	Complete Integrons	In0-Designated	In0 (%)	CALIN Copies (Range; Mean)
Site C	28	25	3	10.7	1–24 (7.8)
Site A	3	0	3	100.0	15–15 (15.0)
Site B	8	8	0	0.0	1–23 (10.9)
Total	39	33	6	15.4	1–24 (8.9)

Fisher’s exact test for In0 prevalence by site: *p* = 0.003 (sample-level, interpreted descriptively given the clonal relatedness of the three Site A isolates). CALIN detected in all 39 isolates (100%).

**Table 9:** VpaIntIA amino-acid variants in six In0-designated isolates.

Variant	Identity to AAK02076.1 (%)	aa Differences	Isolate(s)	ST	Site
A	99.38	2	24-10-07, 24-10-08, 24-10-09	ST4149	Site A
B	99.69	1 (L → I, conservative)	24-06-02	ST1856	Site C
C	99.69	1 (N → S, Block 5)	24-08-05	ST4396	Site C
D	98.75	4 (including R192Q; outside the catalytic-site residues evaluated)	24-07-02	ST4567	Site C

Reference: VpaIntIA from *V. parahaemolyticus* CIP 75.2T (AAK02076.1) [13]. All variants are chromosomal super-integron integrases.

## Data Availability

The 39 *Vibrio parahaemolyticus* genome assemblies generated in this study have been deposited in the DDBJ/ENA/GenBank database under the BioProject accession PRJNA1498204. Novel MLST allelic profiles have been submitted to PubMLST and assigned as ST4566 (isolate 24-06-04), ST4567 (isolate 24-07-02), ST4565 (isolate 24-07-05), and ST4396 (isolate 24-08-05).

## Supplementary Materials

The following supporting information can be downloaded at https://www.mdpi.com/article/10.3390/microorganisms14081846/s1, Table S1: Per-isolate genome quality, ANI, MLST, AMR, integron, CALIN, and prophage features; Table S2: Meteorological characteristics during the 30 days preceding each sampling date; Table S3: VpaIntIA integrase family typing results for In0-designated isolates; Table S4: Integrase family typing workflow parameters and reference sequences.
