# Supplementary material for: Seasonal Occurrence, Population Diversity, and Mobilome Features of Environmental Vibrio parahaemolyticus in Coastal and Estuarine Waters of Haiyan, Zhejiang, China
Source: Microorganisms. 2026 Aug 20;14(8):1846. doi: 10.3390/microorganisms14081846 (PMC13515816; doi:10.3390/microorganisms14081846)
Supplement: Supplementary file 1 [file microorganisms-14-01846-s001.zip › microorganisms-4413123-supplementary.pdf]

Supplementary Table S1. Per-isolate genome quality, ANI, MLST, AMR, integron, CALIN, and prophage features.

Isolate	Site	Month	Genome size (bp)	GC (%)	Contigs	N50 (bp)	Compl. (%)	Contam. (%)	ANI (%)	ST	CARB	Integron type	CALIN	Prophage (kb)
24-06-01	C	Jun	5,136,310	45.23	47	397,876	98.59	1.07	98.30	ST504	CARB-21	Complete	11	33.1
24-06-02	C	Jun	5,547,007	45.47	70	448,528	98.53	3.73	98.20	ST1856	CARB-21	In0	10	131.6
24-06-03	C	Jun	5,306,239	45.17	39	401,716	99.85	1.63	98.29	ST4145	CARB-23	Complete	9	91.1
24-06-04	C	Jun	5,229,977	45.08	39	459,956	98.43	1.38	98.33	ST4566[a]	CARB-23	Complete	8	0.0
24-06-05	C	Jun	5,246,835	45.15	31	430,158	98.83	1.13	98.29	ST477	CARB-21	Complete	6	50.0
24-07-01	C	Jul	5,027,151	45.35	31	494,578	98.66	1.11	98.38	ST2423	CARB-21	Complete	2	66.7
24-07-02	C	Jul	5,545,801	45.37	72	332,565	98.69	3.05	98.21	ST4567[a]	CARB-23	In0	9	121.7
24-07-03	C	Jul	5,054,024	45.35	31	355,776	98.50	1.70	98.23	ST2954	CARB-21	Complete	4	57.0
24-07-04	C	Jul	5,030,104	45.35	31	520,040	98.66	1.11	98.39	ST2423	CARB-21	Complete	2	69.2
24-07-05	C	Jul	5,394,412	45.38	48	299,711	98.67	2.50	98.34	ST4565[a]	CARB-23	Complete	3	143.6
24-08-01	C	Aug	5,131,710	45.29	28	567,132	98.69	1.63	98.32	ST284	CARB-23	Complete	1	45.6
24-08-02	C	Aug	5,131,399	45.29	29	565,593	98.69	1.63	98.27	ST284	CARB-23	Complete	1	45.6
24-08-03	C	Aug	5,365,834	45.21	71	316,561	98.73	2.35	98.30	ST2636	CARB-21	Complete	19	90.7
24-08-04	C	Aug	5,130,803	45.29	32	351,179	98.69	1.63	98.31	ST284	CARB-23	Complete	3	45.6
24-08-05	C	Aug	5,532,743	45.27	77	307,563	98.73	2.73	98.31	ST4396[a]	CARB-23	In0	14	232.9
24-08-06	C	Aug	5,010,423	45.37	37	391,315	98.41	0.68	98.31	ST2137	CARB-21	Complete	8	91.8
24-08-07	B	Aug	5,230,741	45.20	33	387,977	98.71	1.24	98.39	ST169	CARB-23	Complete	5	63.9
24-08-08	B	Aug	5,383,289	45.26	98	207,541	98.69	2.35	98.25	ST2636	CARB-21	Complete	23	91.4
24-09-01	C	Sep	5,262,560	45.15	41	391,296	98.83	1.29	98.32	ST477	CARB-21	Complete	7	50.0
24-09-02	C	Sep	5,012,283	45.31	40	393,144	97.57	1.28	98.25	ST4111	CARB-23	Complete	6	36.0
24-09-03	C	Sep	5,219,577	45.42	41	459,202	98.68	1.51	98.27	ST4110	CARB-21	Complete	7	166.7
24-09-04	C	Sep	5,003,421	45.35	54	460,850	98.72	0.74	98.32	ST4112	CARB-21	Complete	24	78.5
24-09-05	C	Sep	6,103,849	44.02	41	313,440	98.67	1.57	98.33	ST169	CARB-23	Complete	3	132.2
24-09-06	C	Sep	5,982,502	44.05	52	209,338	98.69	0.86	98.30	ST154	CARB-23	Complete	4	158.8
24-09-07	B	Sep	5,127,718	45.35	43	450,852	98.45	1.31	98.29	ST4113	CARB-21	Complete	7	74.8
24-09-08	B	Sep	5,012,332	45.35	29	423,897	98.73	1.29	98.35	ST1815	CARB-23	Complete	4	98.6
24-09-09	B	Sep	4,928,715	45.42	35	511,806	98.73	0.85	98.38	ST942	CARB-21	Complete	11	58.9
24-10-01	C	Oct	5,312,277	45.32	44	423,554	98.42	2.18	98.18	ST4138	CARB-21	Complete	10	138.1
24-10-02	C	Oct	5,248,777	45.15	34	459,727	98.83	1.13	98.29	ST477	CARB-21	Complete	6	50.0
24-10-03	C	Oct	5,248,160	45.15	32	459,727	98.83	1.13	98.28	ST477	CARB-21	Complete	6	50.0
24-10-04	C	Oct	5,023,251	45.32	30	526,007	98.68	1.56	98.30	ST3098	CARB-21	Complete	9	7.8
24-10-05	C	Oct	5,027,694	45.39	33	444,295	98.69	0.94	99.43	ST2251	CARB-21	Complete	4	81.1
24-10-06	C	Oct	5,317,757	45.10	60	349,856	98.86	2.07	98.32	ST4139	CARB-21	Complete	21	25.0
24-10-07	A	Oct	5,055,015	45.26	51	371,234	98.45	1.09	98.29	ST4149	CARB-23	In0	15	43.8
24-10-08	A	Oct	5,057,532	45.26	48	371,234	98.45	1.09	98.27	ST4149	CARB-23	In0	15	43.8
24-10-09	A	Oct	5,056,060	45.26	51	371,234	98.45	1.09	98.30	ST4149	CARB-23	In0	15	43.8
24-10-10	B	Oct	5,359,584	44.89	69	315,501	98.67	1.09	98.25	ST4115	CARB-23	Complete	15	8.9
24-10-11	B	Oct	5,137,084	45.34	25	577,868	98.45	1.09	98.33	ST890	CARB-23	Complete	1	194.2
24-10-12	B	Oct	5,318,000	45.10	56	406,676	98.86	2.07	98.32	ST4139	CARB-21	Complete	21	25.0

[a] Novel STs formally assigned by PubMLST. Site C, coastal seawater; Site A/B, estuarine sites. Compl., CheckM completeness; Contam., CheckM contamination.

Supplementary Table S2. Meteorological characteristics during the 30 days preceding each sampling date.

Sampling Date	Reference Period	Mean Temp (°C)	Mean Max (°C)	Mean Min (°C)	Extreme Max (°C)	Extreme Min (°C)	Total Precip. (mm)	Rainy Days
2024-05-09	Apr 9–May 8	20.0	26	14	31	7	43.0	7
2024-06-18	May 19–Jun 17	24.0	27	21	35	16	112.5	17
2024-07-02	Jun 2–Jul 1	24.0	27	21	35	16	112.5	17
2024-08-27	Jul 28–Aug 26	30.0	34	26	39	24	185.0	15
2024-09-12	Aug 13–Sep 11	28.0	32	24	37	21	168.0	14
2024-10-15	Sep 15–Oct 14	23.0	27	19	33	15	96.0	10

Supplementary Table S3. VpaIntIA integrase family typing results for In0-designated isolates.

Isolate ID	Site	Month	ST	Top BLASTp Hit	Identity vs. AAK02076.1 (%)	VpaIntIA Variant	Interpretation
24-06-02	C	June	ST1856	WP_140116166.1	99.69	Variant B	Chromosomal super-integron integrase
24-07-02	C	July	ST4567	WP_140116166.1	98.75	Variant D	Chromosomal super-integron integrase
24-08-05	C	August	ST4396	WP_140116166.1	99.69	Variant C	Chromosomal super-integron integrase
24-10-07	A	October	ST4149	WP_140116166.1	99.38	Variant A	Chromosomal super-integron integrase
24-10-08	A	October	ST4149	WP_140116166.1	99.38	Variant A	Chromosomal super-integron integrase
24-10-09	A	October	ST4149	WP_140116166.1	99.38	Variant A	Chromosomal super-integron integrase

Supplementary Table S4. Integrase family typing workflow parameters and reference sequences

Category	Item	Details
Reference sequences	VpaIntIA	AAK02076.1 — *Vibrio parahaemolyticus* chromosomal super-integron integrase (320 aa)
	IntI1	AAQ16665.1 — class 1 mobile integron integrase (337 aa)
	IntI2	AAT72891.1 — class 2 mobile integron integrase (325 aa)
	IntI3	AAO32355.1 — class 3 mobile integron integrase (346 aa)
	VchIntIA	AAC38424.1 — Vibrio cholerae chromosomal super-integron integrase (320 aa)
BLASTp parameters	Program	BLAST+ 2.17.0 (blastp)
	Database	Custom reference database built from the 5 sequences above (makeblastdb, protein)
	Scoring matrix	BLOSUM62
	Gap penalties	11 (open), 1 (extend)
	E-value threshold	10 (BLAST+ blastp default; no –evalue flag was set in the run command). All 39 assignment-relevant best hits (VpaIntIA) returned E-values displayed as 0.0 (below BLAST+ reporting precision)
	Output format	Tabular, 12 columns (outfmt 6)
Assignment criteria	Best hit	Reference sequence with the highest percent identity (pident) among the five reference integrases; predefined rule: amino-acid identity > 80%
	Unambiguous	Best hit to a single reference family meeting the > 80% identity rule
	Unresolved	Low identity, short or partial ORF, or near-tie between the top two reference families; flagged for evaluation against a broader reference collection and phylogenetic analysis. Not triggered in this dataset
	Result	39/39 integrases assigned to VpaIntIA (observed identity 95.9–100.0%; mean 99.2%)
Extraction workflow	IntegronFinder version	2.0.5 (Galaxy Pasteur)
	Parameters	–local-max –promoter-attI –dt 4000 –calin-threshold 2 –max-attc-size 200 –min-attc-size 40 –gbk
	ORF extraction	One representative integrase ORF per isolate was extracted from the IntegronFinder GenBank output (stop codon removed); complete integron for 33 isolates and In0 element (integrase without adjacent attC sites) for 6 isolates (24-06-02, 24-07-02, 24-08-05, 24-10-07, 24-10-08, 24-10-09)
Analysis date	BLAST+ verification run	24 July 2026
	NCBI accession verification	2 August 2026

Note: VpaIntIA is the super-integron integrase specific to Vibrio parahaemolyticus; IntI1 – IntI3 represent the canonical mobile class 1 – 3 integron integrases; VchIntIA is the corresponding *V. cholerae* super-integron integrase, included for comparison.
